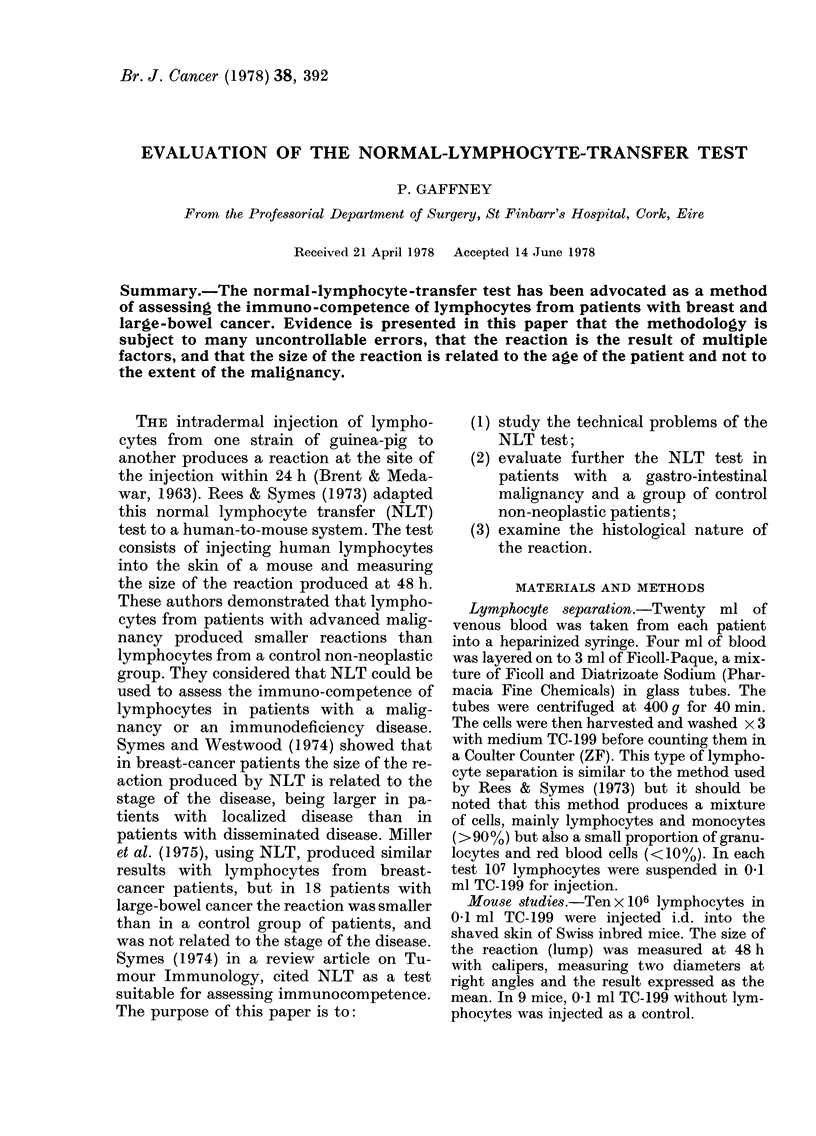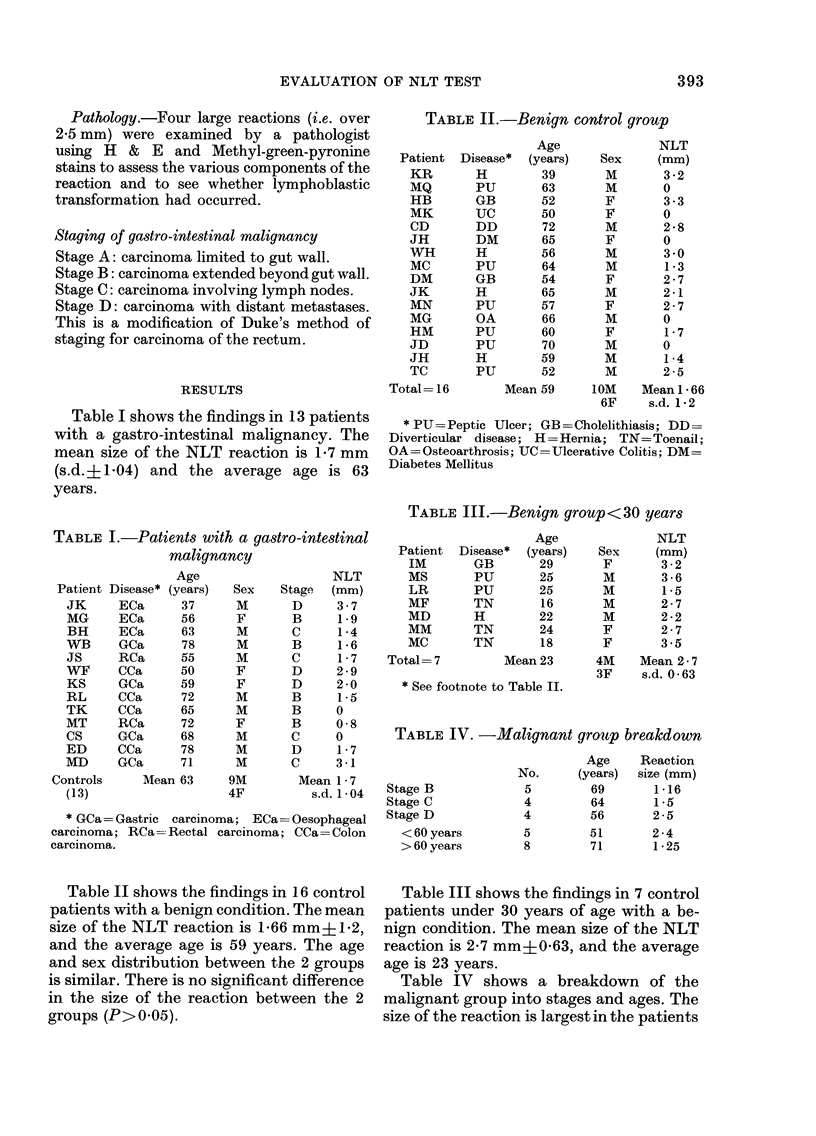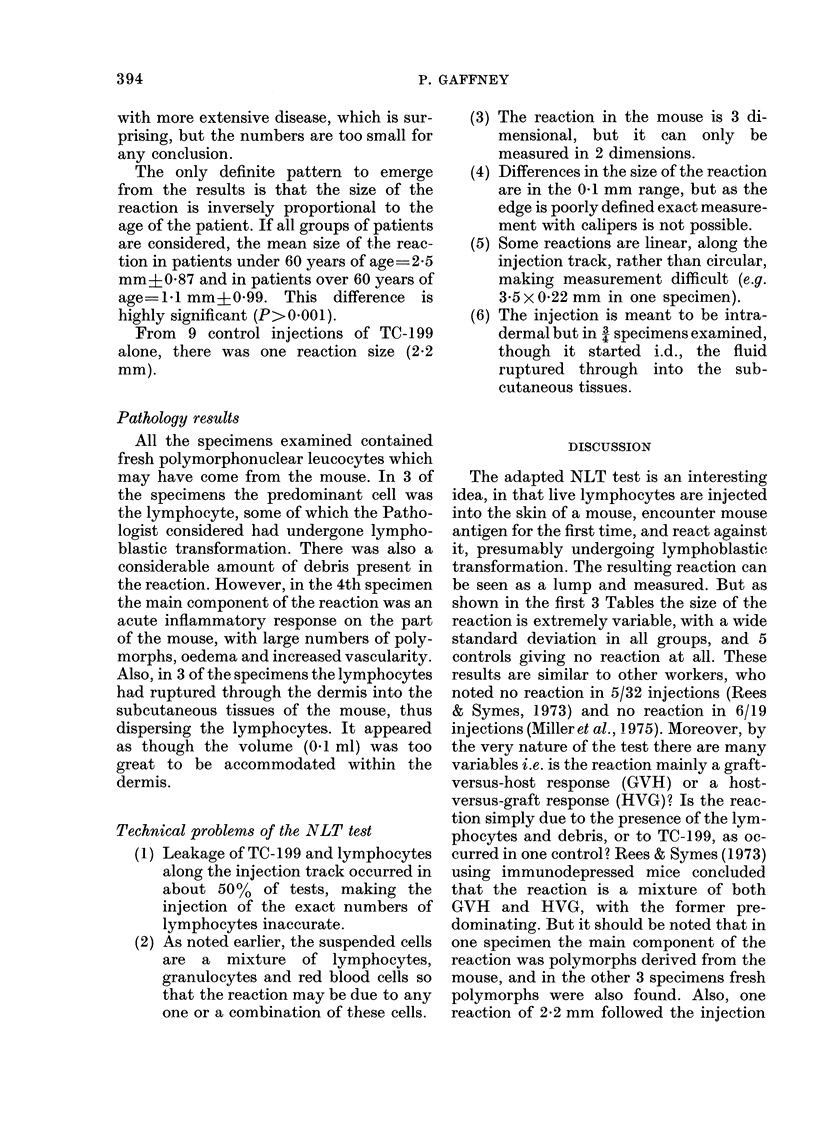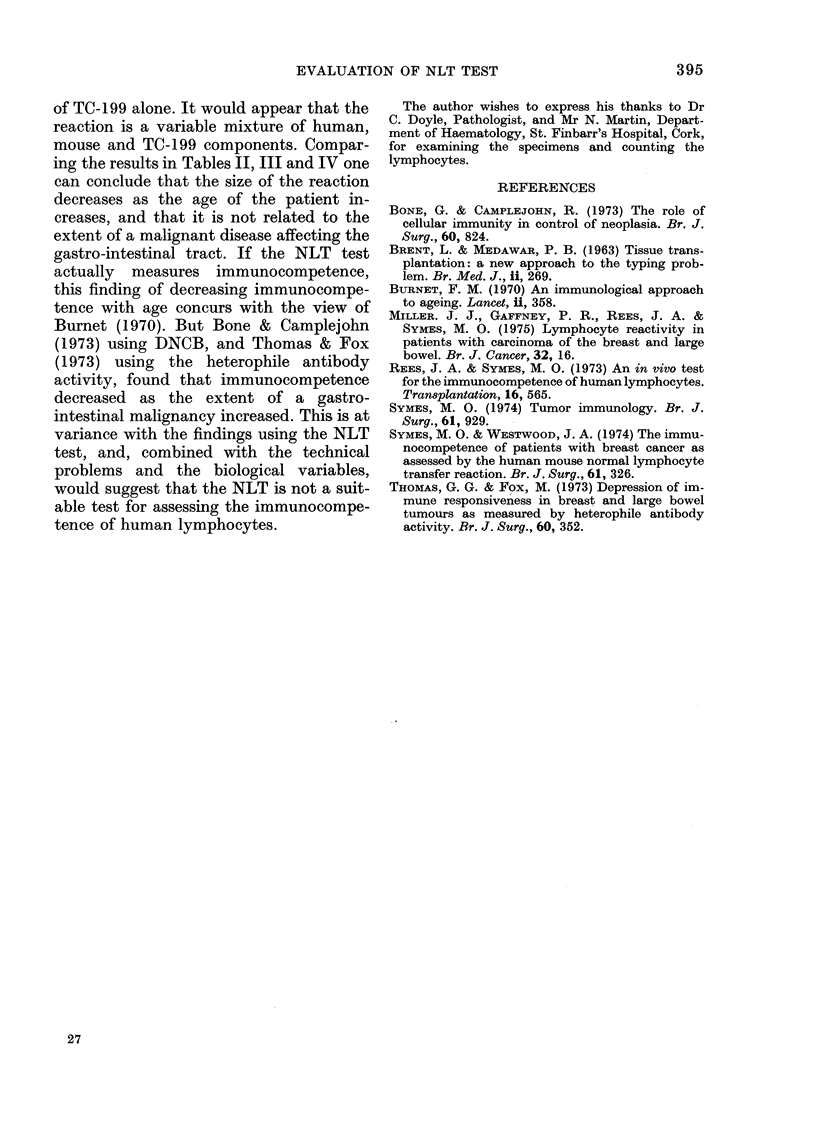# Evaluation of the normal-lymphocyte-transfer test.

**DOI:** 10.1038/bjc.1978.219

**Published:** 1978-09

**Authors:** P. Gaffney

## Abstract

The normal-lymphocyte-transfer test has been advocated as a method of assessing the immuno-competence of lymphocytes from patients with breast and large-bowel cancer. Evidence is presented in this paper that the methodology is subject to many uncontrollable errors, that the reaction is the result of multiple factors, and that the size of the reaction is related to the age of the patient and not to the extent of the malignancy.


*Br. J. Cancer* (1978) **38**, 392

### EVALUATION OF THE NORMAL-LYMPHOCYTE-TRANSFER TEST

P. GAFFNEY

*From the Professorial Department of Surgery, St Finbarr's Hospital, Cork, Eire*

Received 21 April 1978 Accepted 14 June 1978

**Summary.—The normal-lymphocyte-transfer test has been advocated as a method of assessing the immuno-competence of lymphocytes from patients with breast and large-bowel cancer. Evidence is presented in this paper that the methodology is subject to many uncontrollable errors, that the reaction is the result of multiple factors, and that the size of the reaction is related to the age of the patient and not to the extent of the malignancy.**

THE intradermal injection of lymphocytes from one strain of guinea-pig to another produces a reaction at the site of the injection within 24 h (Brent & Medawar, 1963). Rees & Symes (1973) adapted this normal lymphocyte transfer (NLT) test to a human-to-mouse system. The test consists of injecting human lymphocytes into the skin of a mouse and measuring the size of the reaction produced at 48 h. These authors demonstrated that lymphocytes from patients with advanced malignancy produced smaller reactions than lymphocytes from a control non-neoplastic group. They considered that NLT could be used to assess the immuno-competence of lymphocytes in patients with a malignancy or an immunodeficiency disease. Symes and Westwood (1974) showed that in breast-cancer patients the size of the reaction produced by NLT is related to the stage of the disease, being larger in patients with localized disease than in patients with disseminated disease. Miller *et al.* (1975), using NLT, produced similar results with lymphocytes from breast-cancer patients, but in 18 patients with large-bowel cancer the reaction was smaller than in a control group of patients, and was not related to the stage of the disease. Symes (1974) in a review article on Tumour Immunology, cited NLT as a test suitable for assessing immunocompetence. The purpose of this paper is to:

(1) study the technical problems of the NLT test;
(2) evaluate further the NLT test in patients with a gastro-intestinal malignancy and a group of control non-neoplastic patients;
(3) examine the histological nature of the reaction.

### MATERIALS AND METHODS

*Lymphocyte separation.*—Twenty ml of venous blood was taken from each patient into a heparinized syringe. Four ml of blood was layered on to 3 ml of Ficoll-Paque, a mixture of Ficoll and Diatrizoate Sodium (Pharmacia Fine Chemicals) in glass tubes. The tubes were centrifuged at 400 *g* for 40 min. The cells were then harvested and washed $\times 3$ with medium TC-199 before counting them in a Coulter Counter (ZF). This type of lymphocyte separation is similar to the method used by Rees & Symes (1973) but it should be noted that this method produces a mixture of cells, mainly lymphocytes and monocytes ($>90\%$) but also a small proportion of granulocytes and red blood cells ($<10\%$). In each test $10^7$ lymphocytes were suspended in 0·1 ml TC-199 for injection.

*Mouse studies.*—Ten $\times 10^6$ lymphocytes in 0·1 ml TC-199 were injected i.d. into the shaved skin of Swiss inbred mice. The size of the reaction (lump) was measured at 48 h with calipers, measuring two diameters at right angles and the result expressed as the mean. In 9 mice, 0·1 ml TC-199 without lymphocytes was injected as a control.

*Pathology.*—Four large reactions (*i.e.* over 2·5 mm) were examined by a pathologist using H & E and Methyl-green-pyronine stains to assess the various components of the reaction and to see whether lymphoblastic transformation had occurred.

*Staging of gastro-intestinal malignancy*

Stage A: carcinoma limited to gut wall.
Stage B: carcinoma extended beyond gut wall.
Stage C: carcinoma involving lymph nodes.
Stage D: carcinoma with distant metastases.
This is a modification of Duke's method of staging for carcinoma of the rectum.

### RESULTS

Table I shows the findings in 13 patients with a gastro-intestinal malignancy. The mean size of the NLT reaction is 1·7 mm (s.d. $\pm$1·04) and the average age is 63 years.

TABLE I.—*Patients with a gastro-intestinal malignancy*

| Patient | Disease* | Age (years) | Sex | Stage | NLT (mm) |
|---|---|---|---|---|---|
| JK | ECa | 37 | M | D | 3·7 |
| MG | ECa | 56 | F | B | 1·9 |
| BH | ECa | 63 | M | C | 1·4 |
| WB | GCa | 78 | M | B | 1·6 |
| JS | RCa | 55 | M | C | 1·7 |
| WF | CCa | 50 | F | D | 2·9 |
| KS | GCa | 59 | F | D | 2·0 |
| RL | CCa | 72 | M | B | 1·5 |
| TK | CCa | 65 | M | B | 0 |
| MT | RCa | 72 | F | B | 0·8 |
| CS | GCa | 68 | M | C | 0 |
| ED | CCa | 78 | M | D | 1·7 |
| MD | GCa | 71 | M | C | 3·1 |
| Controls (13) | | Mean 63 | 9M 4F | | Mean 1·7 s.d. 1·04 |

* GCa = Gastric carcinoma; ECa = Oesophageal carcinoma; RCa = Rectal carcinoma; CCa = Colon carcinoma.

TABLE II.—*Benign control group*

| Patient | Disease* | Age (years) | Sex | NLT (mm) |
|---|---|---|---|---|
| KR | H | 39 | M | 3·2 |
| MQ | PU | 63 | M | 0 |
| HB | GB | 52 | F | 3·3 |
| MK | UC | 50 | F | 0 |
| CD | DD | 72 | M | 2·8 |
| JH | DM | 65 | F | 0 |
| WH | H | 56 | M | 3·0 |
| MC | PU | 64 | M | 1·3 |
| DM | GB | 54 | F | 2·7 |
| JK | H | 65 | M | 2·1 |
| MN | PU | 57 | F | 2·7 |
| MG | OA | 66 | M | 0 |
| HM | PU | 60 | F | 1·7 |
| JD | PU | 70 | M | 0 |
| JH | H | 59 | M | 1·4 |
| TC | PU | 52 | M | 2·5 |
| Total = 16 | | Mean 59 | 10M 6F | Mean 1·66 s.d. 1·2 |

* PU = Peptic Ulcer; GB = Cholelithiasis; DD = Diverticular disease; H = Hernia; TN = Toenail; OA = Osteoarthrosis; UC = Ulcerative Colitis; DM = Diabetes Mellitus

TABLE III.—*Benign group* $<$ 30 *years*

| Patient | Disease* | Age (years) | Sex | NLT (mm) |
|---|---|---|---|---|
| IM | GB | 29 | F | 3·2 |
| MS | PU | 25 | M | 3·6 |
| LR | PU | 25 | M | 1·5 |
| MF | TN | 16 | M | 2·7 |
| MD | H | 22 | M | 2·2 |
| MM | TN | 24 | F | 2·7 |
| MC | TN | 18 | F | 3·5 |
| Total = 7 | | Mean 23 | 4M 3F | Mean 2·7 s.d. 0·63 |

* See footnote to Table II.

TABLE IV. —*Malignant group breakdown*

| | No. | Age (years) | Reaction size (mm) |
|---|---|---|---|
| Stage B | 5 | 69 | 1·16 |
| Stage C | 4 | 64 | 1·5 |
| Stage D | 4 | 56 | 2·5 |
| $<$ 60 years | 5 | 51 | 2·4 |
| $>$ 60 years | 8 | 71 | 1·25 |

Table II shows the findings in 16 control patients with a benign condition. The mean size of the NLT reaction is 1·66 mm$\pm$1·2, and the average age is 59 years. The age and sex distribution between the 2 groups is similar. There is no significant difference in the size of the reaction between the 2 groups ($P > 0{\cdot}05$).

Table III shows the findings in 7 control patients under 30 years of age with a benign condition. The mean size of the NLT reaction is 2·7 mm$\pm$0·63, and the average age is 23 years.

Table IV shows a breakdown of the malignant group into stages and ages. The size of the reaction is largest in the patients with more extensive disease, which is surprising, but the numbers are too small for any conclusion.

The only definite pattern to emerge from the results is that the size of the reaction is inversely proportional to the age of the patient. If all groups of patients are considered, the mean size of the reaction in patients under 60 years of age$=2 \cdot 5$ mm$\pm 0 \cdot 87$ and in patients over 60 years of age$=1 \cdot 1$ mm$\pm 0 \cdot 99$. This difference is highly significant ($P > 0 \cdot 001$).

From 9 control injections of TC-199 alone, there was one reaction size (2·2 mm).

*Pathology results*

All the specimens examined contained fresh polymorphonuclear leucocytes which may have come from the mouse. In 3 of the specimens the predominant cell was the lymphocyte, some of which the Pathologist considered had undergone lymphoblastic transformation. There was also a considerable amount of debris present in the reaction. However, in the 4th specimen the main component of the reaction was an acute inflammatory response on the part of the mouse, with large numbers of polymorphs, oedema and increased vascularity. Also, in 3 of the specimens the lymphocytes had ruptured through the dermis into the subcutaneous tissues of the mouse, thus dispersing the lymphocytes. It appeared as though the volume (0·1 ml) was too great to be accommodated within the dermis.

*Technical problems of the NLT test*

(1) Leakage of TC-199 and lymphocytes along the injection track occurred in about 50% of tests, making the injection of the exact numbers of lymphocytes inaccurate.
(2) As noted earlier, the suspended cells are a mixture of lymphocytes, granulocytes and red blood cells so that the reaction may be due to any one or a combination of these cells.
(3) The reaction in the mouse is 3 dimensional, but it can only be measured in 2 dimensions.
(4) Differences in the size of the reaction are in the 0·1 mm range, but as the edge is poorly defined exact measurement with calipers is not possible.
(5) Some reactions are linear, along the injection track, rather than circular, making measurement difficult (*e.g.* $3 \cdot 5 \times 0 \cdot 22$ mm in one specimen).
(6) The injection is meant to be intradermal but in $\frac{3}{4}$ specimens examined, though it started i.d., the fluid ruptured through into the subcutaneous tissues.

### DISCUSSION

The adapted NLT test is an interesting idea, in that live lymphocytes are injected into the skin of a mouse, encounter mouse antigen for the first time, and react against it, presumably undergoing lymphoblastic transformation. The resulting reaction can be seen as a lump and measured. But as shown in the first 3 Tables the size of the reaction is extremely variable, with a wide standard deviation in all groups, and 5 controls giving no reaction at all. These results are similar to other workers, who noted no reaction in 5/32 injections (Rees & Symes, 1973) and no reaction in 6/19 injections (Miller *et al.*, 1975). Moreover, by the very nature of the test there are many variables *i.e.* is the reaction mainly a graft-versus-host response (GVH) or a host-versus-graft response (HVG)? Is the reaction simply due to the presence of the lymphocytes and debris, or to TC-199, as occurred in one control? Rees & Symes (1973) using immunodepressed mice concluded that the reaction is a mixture of both GVH and HVG, with the former predominating. But it should be noted that in one specimen the main component of the reaction was polymorphs derived from the mouse, and in the other 3 specimens fresh polymorphs were also found. Also, one reaction of 2·2 mm followed the injection of TC-199 alone. It would appear that the reaction is a variable mixture of human, mouse and TC-199 components. Comparing the results in Tables II, III and IV one can conclude that the size of the reaction decreases as the age of the patient increases, and that it is not related to the extent of a malignant disease affecting the gastro-intestinal tract. If the NLT test actually measures immunocompetence, this finding of decreasing immunocompetence with age concurs with the view of Burnet (1970). But Bone & Camplejohn (1973) using DNCB, and Thomas & Fox (1973) using the heterophile antibody activity, found that immunocompetence decreased as the extent of a gastro-intestinal malignancy increased. This is at variance with the findings using the NLT test, and, combined with the technical problems and the biological variables, would suggest that the NLT is not a suitable test for assessing the immunocompetence of human lymphocytes.

The author wishes to express his thanks to Dr C. Doyle, Pathologist, and Mr N. Martin, Department of Haematology, St. Finbarr's Hospital, Cork, for examining the specimens and counting the lymphocytes.

### REFERENCES

BONE, G. & CAMPLEJOHN, R. (1973) The role of cellular immunity in control of neoplasia. *Br. J. Surg.*, **60**, 824.

BRENT, L. & MEDAWAR, P. B. (1963) Tissue transplantation: a new approach to the typing problem. *Br. Med. J.*, **ii**, 269.

BURNET, F. M. (1970) An immunological approach to ageing. *Lancet*, **ii**, 358.

MILLER. J. J., GAFFNEY, P. R., REES, J. A. & SYMES, M. O. (1975) Lymphocyte reactivity in patients with carcinoma of the breast and large bowel. *Br. J. Cancer*, **32**, 16.

REES, J. A. & SYMES, M. O. (1973) An *in vivo* test for the immunocompetence of human lymphocytes. *Transplantation*, **16**, 565.

SYMES, M. O. (1974) Tumor immunology. *Br. J. Surg.*, **61**, 929.

SYMES, M. O. & WESTWOOD, J. A. (1974) The immunocompetence of patients with breast cancer as assessed by the human mouse normal lymphocyte transfer reaction. *Br. J. Surg.*, **61**, 326.

THOMAS, G. G. & FOX, M. (1973) Depression of immune responsiveness in breast and large bowel tumours as measured by heterophile antibody activity. *Br. J. Surg.*, **60**, 352.